# Laccase Biosensor Based on Electrospun Copper/Carbon Composite Nanofibers for Catechol Detection

**DOI:** 10.3390/s140203543

**Published:** 2014-02-20

**Authors:** Jiapeng Fu, Hui Qiao, Dawei Li, Lei Luo, Ke Chen, Qufu Wei

**Affiliations:** Key Laboratory of Eco-textiles, Ministry of Education, Jiangnan University, Wuxi 214122, China; E-Mails: firgexiao@sina.cn (J.F.); huiqiaoz@163.com (H.Q.); ldw19900323@163.com (D.L.); luolei891123@126.com (L.L.); chenke2327@163.com (K.C.)

**Keywords:** electrospinning, copper/carbon composite nanofibers, laccase biosensor, catechol detection, electrochemical properties

## Abstract

The study compared the biosensing properties of laccase biosensors based on carbon nanofibers (CNFs) and copper/carbon composite nanofibers (Cu/CNFs). The two kinds of nanofibers were prepared by electrospinning and carbonization under the same conditions. Scanning electron microscopy (SEM), X-ray diffraction (XRD) and Raman spectroscopy were employed to investigate the morphologies and structures of CNFs and Cu/CNFs. The amperometric results indicated that the Cu/CNFs/laccase(Lac)/Nafion/glass carbon electrode (GCE) possessed reliable analytical performance for the detection of catechol. The sensitivity of the Cu/CNFs/Lac/Nafion/GCE reached 33.1 μA/mM, larger than that of CNFs/Lac/Nafion/GCE. Meanwhile, Cu/CNFs/Lac/Nafion/GCE had a wider linear range from 9.95 × 10^−6^ to 9.76 × 10^−3^ M and a lower detection limit of 1.18 μM than CNFs/Lac/Nafion/GCE. Moreover, it exhibited a good repeatability, reproducibility, selectivity and long-term stability, revealing that electrospun Cu/CNFs have great potential in biosensing.

## 1. Introduction

As one of the more common environmental contaminants, catechol originates mainly from a variety of chemicals and pesticides. Due to its toxicity, methods such as high-performance liquid chromatography [1], spectrophotometric [2] and electrochemical approaches [3] applied in biosensors have been adopted for catechol monitoring. Because of their high selectivity, high sensitivity, simplicity, reliability and rapid online monitoring character, biosensors have received much attention in fundamental science [4,5], environmental monitoring [6] and food quality [7,8]. However, elimination of interferences and the nature of the immobilization matrix, which plays a very important role in the efficiency and signal transduction for improving sensitivity and the stability of the biological sensing element, are still critical issues in biosensor systems [9].

To improve the performance of biosensors, there has been increasing attention paid to nanomaterials [10–13], carbon-based nanomaterials in particular. For example, carbon nanotubes were used for the design of a pyranose oxidase biosensor, which was applied to glucose analysis in wine samples [14]. Similarly, the glucose biosensor based on highly activated carbon nanofibers, which exhibited a very sensitive, stable and reproducible electrochemical performance, indicated that the carbon nanofibers were the best matrix [15]. Carbon-based nanomaterials are conductive, easily functionalized, biocompatible, and possess large surface areas. These characteristics make their various forms, including carbon nanotubes [14,16,17], carbon nanofibers (CNFs) [15,18] and ordered mesoporous carbon [19], ideal for biosensor applications. Compared to carbon nanotubes, the very large surface area of CNFs can be well controlled [9], providing a high-surface immobilization matrix for the entrapment or attachment of biomolecules. At the same time, CNFs can play a role as transducers due to their high conductance [20]. On the other hand, doping carbon-based nanomaterials with metals has been proven to be an efficient method to enhance the sensitivity and stability of the biosensors [9,21,22]. For example, a Pd nanoparticles-decorated graphene oxide prepared by an *in situ* reduction method for a glucose biosensor provided a biocompatible platform for biosensing and biocatalysis [21].

Electrospinning has been most widely employed to produce fibers with diameters ranging from a few nanometers to several micrometers. The carbonization of elctrospun polymer nanofibers and the reduction of metal ion were performed on metal nanoparticle-doped carbon nanofibers [23–26]. Compared with other metal nanoparticles, copper nanoparticles were selected in this paper since they are relatively inexpensive and also have good biocompatibility and electrical conducting properties, resulting in an increased response current [27]. Nafion polymer, due to its attractive properties, can also improve the long-term stability, enhance the adhesion and binding force and the selectivity of a biosensor [28,29]. In addition, the operations for the preparation of Nafion modified electrodes are easy.

In this work, copper nanoparticles were incorporated into CNFs to develop a novel electrochemical enzyme biosensor. The morphologies and structures of CNFs and Cu/CNFs were investigated by scanning electron microscopy (SEM), X-ray diffraction (XRD) and Raman spectroscopy. The biosensing properties of the biosensors based on electrospun CNFs and Cu/CNFs were also studied.

## 2. Experimental Section

### 2.1. Materials

All reagents were used as received without further purification. Polyvinylpyrrolidone (PVP, Mw = 1.3 × 10^6^), N,N-dimethylformamide (DMF), Cu(Ac)_2_·H_2_O, CH_3_COOH, CH_3_COONa, guaiacol, vanillin, phenol and 3,5-dinitrosalicylic acid were purchased from Sinopharm Chemical Reagent Co., Ltd. (Shanghai, China). Polyacrylonitrile (PAN, Mw = 50,000–60,000) was purchased from Zhejiang Shangyu Wuyue Trade Co., Ltd. (Shangyu, Chaina). Catechol was purchased from Aladdin Reagent Co., Ltd. (Shanghai, China). Laccase (Lac) and Nafion were purchased from Sigma-Aldrich Chemical Co., Ltd. (St. Louis, MO, USA). All water was deionized. A 0.1 M acetate buffer solution was employed as supporting electrolyte.

### 2.2. Preparation of CNFs and Cu/CNFs

The electrospinning method [23,24,30] and carbonization were adopted to fabricate CNFs and Cu/CNFs. PAN solution (10 wt%) was prepared by dissolving PAN (2.045 g) in DMF (19.6 mL). PVP (0.228 g) and Cu(Ac)_2_·H_2_O (1.142 g) were added to PAN solution and stirred for 6 h at about 60 °C to obtain the mixed solution. The weight ratio of PVP to PAN was 1:9. The weight ratio of Cu(Ac)_2_·H_2_O to the two polymers were 0:2 and 1:2, respectively. During the electrospinning process, the precursor solution was placed in a syringe. The positive electrode of the high voltage power supply was connected to the needle tip. The grounded electrode was connected to a drum collector wrapped with an aluminum foil. The flow rate and tip-collector distance were fixed at 0.5 mL/h and 20 cm, respectively. Dry fibers were accumulated on the aluminum foil and collected as a fibrous mat. The as-spun PAN/PVP and PAN/PVP/Cu(Ac)_2_·H_2_O composite nanofibers were later converted into CNFs and Cu/CNFs by pre-oxidizing at 280 °C for 1 h under air atmosphere (a heating rate 2 °C/min) and carbonizing at 900 °C for 2 h under nitrogen atmosphere in a tube furnace (a heating rate 2 °C/min), respectively.

### 2.3. Preparation of CNFs/Lac/Nafion/GCE and Cu/CNFs/Lac/Nafion/GCE

Before modification, the glass carbon electrode (GCE) was polished with 0.05 μm alumina slurry on a polishing cloth, rinsed thoroughly with water and sonicated in water for 5 min. CNFs (6 mg) were dispersed in 0.1 M acetate buffer (10 mL, pH = 4.0) under ultrasonic agitation for 0.5 h. To optimize current response and stability, the concentrations and their mass ratio in nanofibers, the amounts of Lac and Nafion in the mixture were optimized in control experiments. Then Lac (15 mg) and Nafion (140 mg) were added to the CNFs solutions. The mixed solution was stirred for six hours. Mixed solution (10 μL) coated onto the surface of the GCE. Then it was left to dry in refrigerator under 4 °C. The electrode was recognized as CNFs/Lac/Nafion/GCE. CNFs/Lac/Nafion/GCE was stored at 4 °C for later use.

For comparison with CNFs/Lac/Nafion/GCE, Cu/CNFs/Lac/Nafion/GCE was prepared with similar procedures as described above. The solution containing Cu/CNFs (6 mg), laccase (15 mg) and Nafion (140 mg) was used to prepare the Cu/CNFs/Lac/Nafion/GCE.

### 2.4. Characterization of CNFs and Cu/CNFs

The morphology and diameter of both CNFs and Cu/CNFs were characterized using scanning electron microscopy (SEM, S-4800, Hitachi, Tokyo, Japan, at 5 kV). X-ray diffraction (XRD, D8 Advance X-ray Diffraction System, Bruker, Karlsruhe, Germany, Cu Kα, k = 1.5405 Å) and Raman spectroscopy (NEXUS-6700 FTIR-Raman spectrometer, Wisconsin, US, 533 nm HeNe Laser) were employed to examine the crystal and chemical structures of CNFs and Cu/CNFs.

### 2.5. Electrochemical Measurements

All electrochemical measurements were performed using a CHI 660B electrochemical workstation (CH Instruments, Shanghai, China). The electrochemical experiments were carried out using a conventional three-electrode with a glassy carbon working electrode (GCE, 4.0 mm), a platinum wire as the counter electrode, and an Ag/AgCl (saturated KCl) electrode as the reference electrode, respectively. Before electrochemical measurements, all the electrodes were immersed into pH 4.0 acetate buffer for 30 min to remove the residual components. The 0.1 M acetate buffer was used as electrolyte and its volume was equal to 20 mL. All the experiments were performed in batch mode at room temperature.

## 3. Results and Discussions

### 3.1. Morphology and Structure Analysis

**Figure 1.**
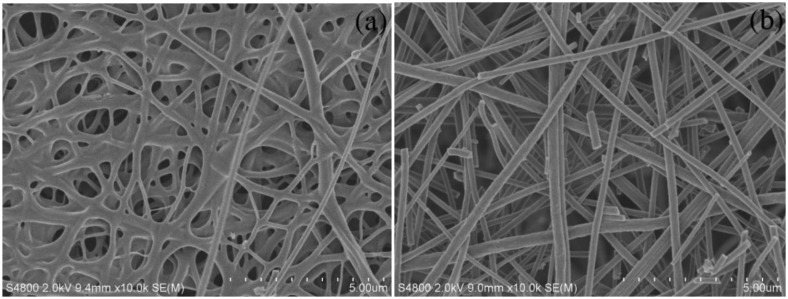
SEM images of (**a**) CNFs and (**b**) Cu/CNFs.

The SEM images of the CNFs and Cu/CNFs are shown in Figure 1. As shown in Figure 1a, the obtained CNFs presented a fibrous structure in most parts and some fiber-fiber interconnections at the intersection areas. The inter-fiber connection was formed from the carbonized PVP component [31]. The fiber diameter on the fibrous section varied from 104 to 217 nm, and its average diameter was about 170 nm. In comparison with CNFs, Cu/CNFs were more uniform and had no interconnections. In addition, the diameter distribution of Cu/CNFs ranged from 240 to 340 nm, with an average diameter of 300 nm.

**Figure 2.**
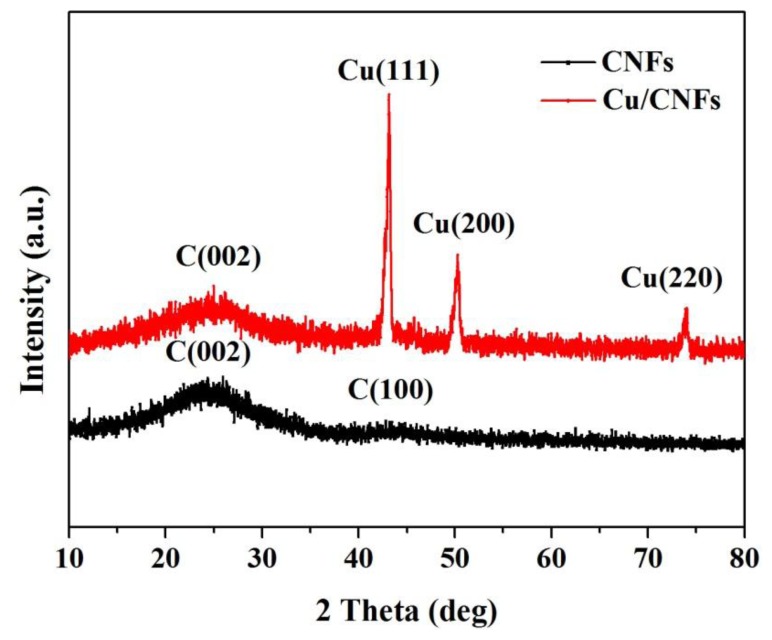
XRD patterns of CNFs and Cu/CNFs.

Figure 2 presents the XRD patterns of CNFs and Cu/CNFs. It was observed that the pure CNFs exhibited two broad and weak diffraction peaks at 2θ = 24° and 43°, corresponding to the graphitic crystallite planes (002) and (100), respectively [32]. Unlike the pure CNFs, three diffraction peaks at 2θ = 44°, 51° and 74° were detected, which corresponded to the diffraction peaks of (111), (200) and (220) of Cu (JCPDS NO.04-0836). These diffraction peaks were narrow and sharp. The XRD pattern indicated that copper was well crystallized and cubic in structure. Based on the Scherrer equation, the crystallite size of copper nanoparticle in the composite fibers obtained at 900 °C was 20.8 nm. The XRD results confirmed the existence of carbon and copper in the composite nanofibers.

**Figure 3.**
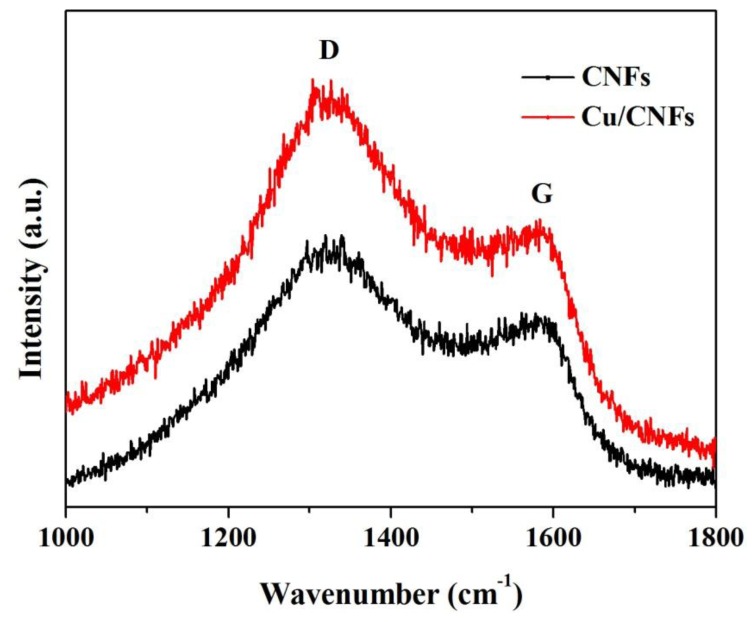
Raman spectrum of CNFs and Cu/CNFs.

Raman spectroscopy has extensively been used as a surface analysis technique for characterization of carbon-containing materials [33,34]. The Raman spectra from 1,000 to 1,800 cm^−1^ of CNFs and Cu/CNFs are shown in Figure 3. The two materials exhibited two distinct peaks at about 1,350 cm^−1^ and 1,580 cm^−1^, which corresponded to the D, and G bands, respectively. The D bond was referred to the “disorder” band, which resulted from defects and the edge of the carbon nanofibers [35,36]. The relative intensity ratio between the D and G bands indicated that these nanofibers contained a lot of defects and edge sites.

### 3.2. Direct Electrochemistry and Electrocatalysis of the Biosensors

**Figure 4.**
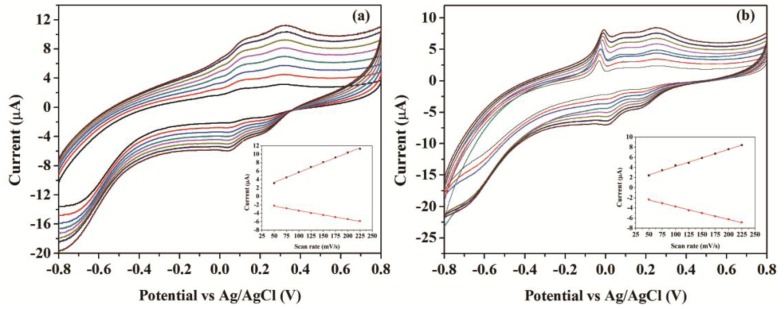
CVs of (**a**) CNFs/Lac/Nafion/GCE and (**b**) Cu/CNFs/Lac/Nafion/GCE in 0.1 M, pH 4.0, acetate buffer solutions at scan rates from the inner to the outer: 50, 75, 100, 125, 150, 175, 200, 225 mV/s. Insets: calibration plots of anodic and cathodic peak currents *vs.* scan rates.

Figure 4 presents the cyclic voltammograms (CVs) of the CNFs/Lac/Nafion/GCE and Cu/CNFs/Lac/Nafion/GCE in pH 4.0 acetate buffer solutions with scan rates from 50 to 225 mV/s. From Figure 4a, it can be clearly seen that a pair of stable and well-defined quasi-reversible anodic and cathodic peaks were presented, which could be attributed to the direct electron transfer between laccase and GCE. The cathodic and anodic peak currents increased linearly with the increase of the scan rates in that range (insets in Figure 4). This revealed that the electron transfer between laccase and GCE was a quasi-reversible surface control process. Unlike CNFs/Lac/Nafion/GCE, Cu/CNFs/Lac/Nafion/GCE exhibited a sharp peak at the potential of 0 V (Figure 4b). It can be ascribed to the oxidation of copper. Compared with the ratio of Ipc to Ipa of 0.427 at CNFs/Lac/Nafion/GCE, the ratio of 0.757 at Cu/CNFs/Lac/Nafion/GCE was bigger, indicating a better reversibility at Cu/CNFs/Lac/Nafion/GCE.

### 3.3. Optimization of the Biosensors Operating Conditions

The response of the modified electrode is affected by several parameters such as pH, operating potential, enzyme loading of the matrix, and temperature. In this study, pH and operating potential were optimized for the detection of catechol by amperometric measurements.

**Figure 5.**
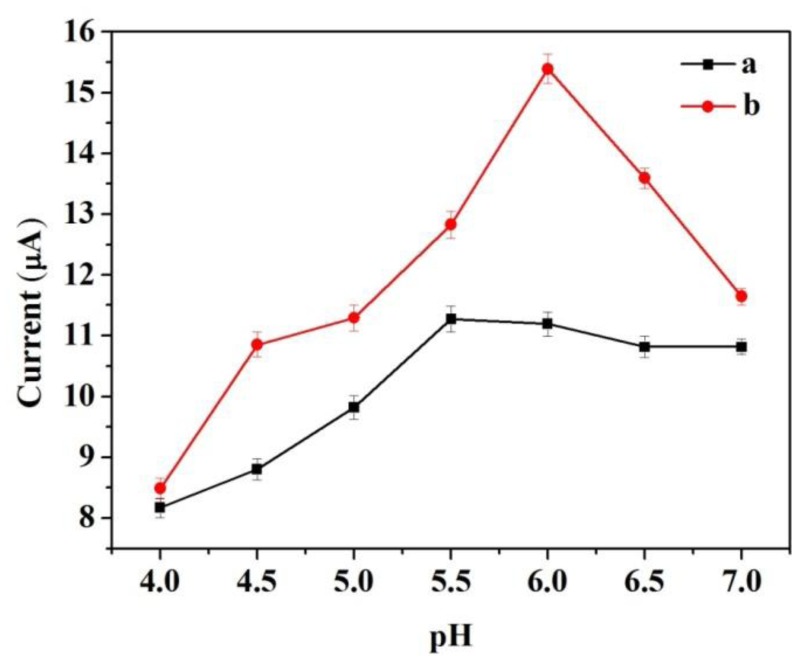
The dependence of the response of (**a**) CNFs/Lac/Nafion/GCE and (**b**) Cu/CNFs/Lac/Nafion/GCE to an input of 3.92 × 10^−4^ M catechol on the pH of the buffer. Conditions: operating potential, 0.5 V *versus* Ag/AgCl reference. The error bars indicate the standard error for triplicate measurements with the same electrode.

To determine the optimum pH for detecting catechol at the modified electrode, control experiments were performed to compare the amperometric response for the same concentration of catechol at different pH values ranging from 4.0 to 7.0. The two modified electrodes were tested. Figure 5 shows the response to an input of 3.92 × 10^−4^ M catechol. The two modified electrodes did not display a broad pH optimum. To some extent, the response increased with increasing pH and decreased at higher pH. The largest amperometric response of CNFs/Lac/Nafion/GCE was achieved at pH 5.5. However, at Cu/CNFs/Lac/Nafion/GCE, an optimum pH of 6.0 was noted, which was also found as optimum for laccase in other matrixes [27]. The differences in pH optimum between the two electrodes may be attributed to effects of different matrixes on the physicochemical properties of the laccase.

**Figure 6.**
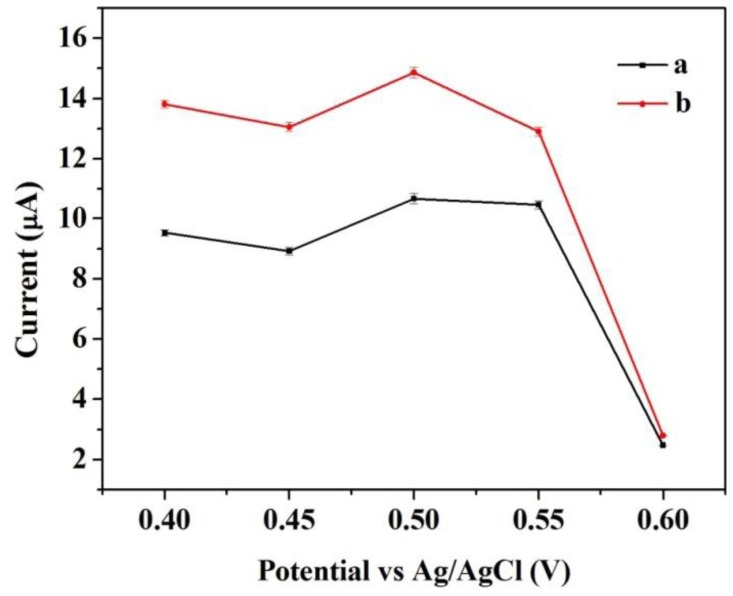
The dependence of the response of (**a**) CNFs/Lac/Nafion/GCE and (**b**) Cu/CNFs/Lac/Nafion/GCE to an input of 3.92 × 10^−4^ M catechol on the operating potential. Conditions: pH 5.5 (a), pH 6.0 (b). The error bars indicate the standard error for triplicate measurements with the same electrode.

Similarly, control experiments were also carried out to compare the amperometric response for the same concentration of catechol at different operating potential varying from 0.4 to 0.6 V. Figure 6 shows the response to an input of 3.92 × 10^−4^ M catechol. The two modified electrodes presented the same trend towards the change in operating potential. The response reached a peak at operating potential of 0.5 V *versus* Ag/AgCl reference. The initial signal became unstable with higher operating potential, which may be caused by possible interferences. Thus, the operating potential of 0.5 V *versus* Ag/AgCl reference was selected in the further experiments. It was very obvious that the response of Cu/CNFs/Lac/Nafion/GCE was stronger than that of CNFs/Lac/Nafion/GCE under the same conditions. This will be further discussed in the following sections.

### 3.4. Amperometric Response of the Biosensors

**Figure 7.**
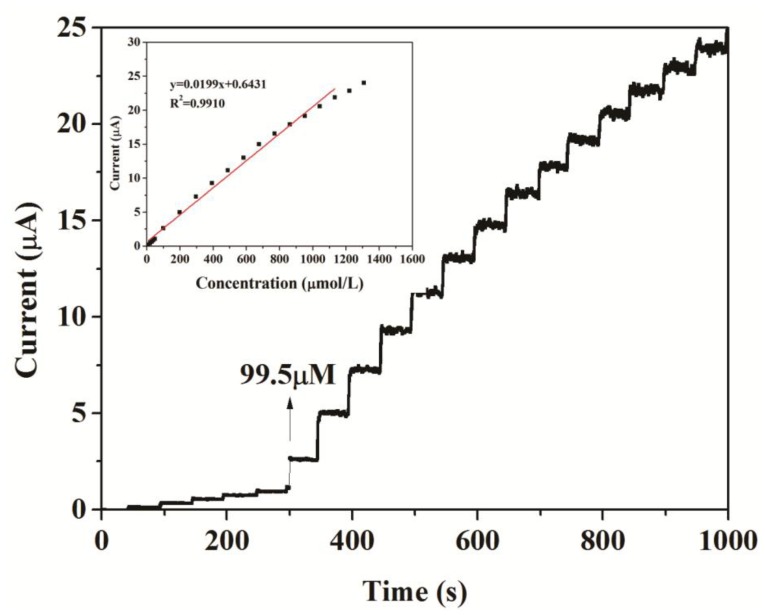
Current-time curve of CNFs/Lac/Nafion/GCE with successive addition of catechol in 0.10 M, pH 5.5, acetate buffer solutions at operating potential of +0.5 V (*vs.* Ag/AgCl). Inset: calibration plot illustrating the linear electrode response to catechol addition.

**Figure 8.**
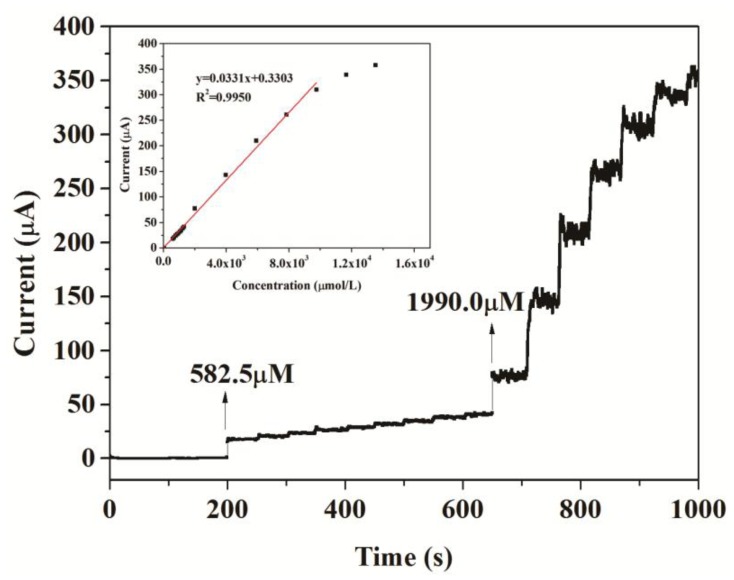
Current-time curve of Cu/CNFs/Lac/Nafion/GCE with successive addition of catechol in 0.10 M, pH 6.0, acetate buffer solutions at operating potential of +0.5 V(*vs*. Ag/AgCl). Inset: calibration plot illustrating the linear electrode response to catechol addition.

Under the optimum conditions established in the section above, the typical response of the two biosensors to successive injections of different volumes of 2 mM , 20 mM and 400 mM catechol into 20 mL acetate buffer at + 0.5 V are shown in Figure 7 and Figure 8, respectively. When catechol was added into buffer solution, the response rose steeply and reached a steady state within 5 s. It suggested that the modified electrodes responded rapidly to the change in the substrate concentration.

The response current of the biosensors responded linearly to catechol at lower concentrations and reached a saturation level at a higher concentration, as expected from Michaelis-Menten type enzyme kinetics (inset in Figure 7). The linear response range of CNFs/Lac/Nafion/GCE was from 9.95 ×10^−6^ to 1.13 × 10^−3^ M. The linear regression equation was y (μA) = 0.6431 + 0.0199x (μM), with a correlation coefficient of 0.9910 (n = 19, inset in Figure 7). As a comparison, the linear response range of Cu/CNFs/Lac/Nafion/GCE was 9.95 × 10^−6^ to 9.76 × 10^−3^ M (y (μA) = 0.3303 + 0.0331x (μM), R^2^ = 0.9950) (Figure 8), which was comparable to that of 9.95 × 10^−6^ – 1.13×10^−3^ M for the CNFs/Lac/Nafion/GCE (inset in Figure 7), 1.2 × 10^−6^ to 3 × 10^−5^ M for the Lac/CNTs–CS/GCE [37], 4.0 × 10^−6^ to 8.8 × 10^−5^ M for the MB-MCM-41/PVA/Lac [38], 6.7 × 10^−7^ to 1.38 × 10^−5^ M for the Cu-OMC/Lac/CS/Au [19], 1.5 × 10^−5^ to 7.0 × 10^−4^ M for the Lac/AP-rGOs/Chit/GCE [39].

Thus, the detection limit of Cu/CNFs/Lac/Nafion/GCE was estimated to be 1.18 μM at a signal/noise (S/N) of 3, which was lower than that of 3.32 μM for the CNFs/Lac/Nafion/GCE. The sensitivity of the Cu/CNFs/Lac/Nafion/GCE to the detection of catechol was 33.1 μA/mM, which was larger than that of 19.9 μA/mM for the CNFs/Lac/Nafion/GCE (Figure 7), 15.8 μA/mM for the Lac/AP-rGOs/Chit/GCE [39]. They exhibited quite outstanding analytical performance.

**Figure 9.**
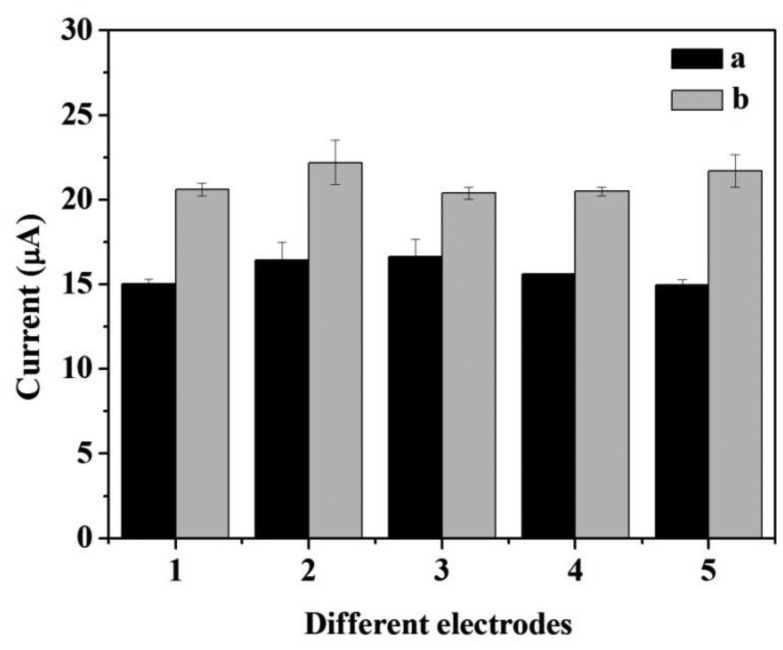
The repeatability and the reproducibility of the response of (**a**) CNFs/Lac/Nafion/GCE and (**b**) Cu/CNFs/Lac/Nafion/GCE. Conditions: scan rate 100 mV/s, pH 5.5 (a), pH 6.0 (b). The error bars indicate the standard error for three measurements with the same electrode.

The repeatability and the reproducibility of the two biosensors are illustrated in Figure 9. The error bars indicated the standard error for triplicate measurements with the same electrode. The relative standard deviation (RSD) of CNFs/Lac/Nafion/GCE and Cu/CNFs/Lac/Nafion/GCE were within 6.53% and 4.35%, respectively, indicating that Cu/CNFs/Lac/Nafion/GCE had better repeatability than CNFs/Lac/Nafion/GCE. The fabrication reproducibility of the two biosensors were calculated by cyclic voltammetric measurements on the same day, using five electrodes which were made independently under the optimum pH conditions previously established and in the presence of 3.92 × 10^−4^ M catechol. A RSD of CNFs/Lac/Nafion/GCE was 4.89% obtained, revealing an acceptable reproducibility in the construction of the electrode. Moreover, Cu/CNFs/Lac/Nafion/GCE with a RSD of 3.79% is better than CNF/Lac/Nafion/GCE in reproducibility. Also, the stability of the electrodes is considered. When the biosensor was not in use, it was stored in buffer at 4 °C.

**Figure 10.**
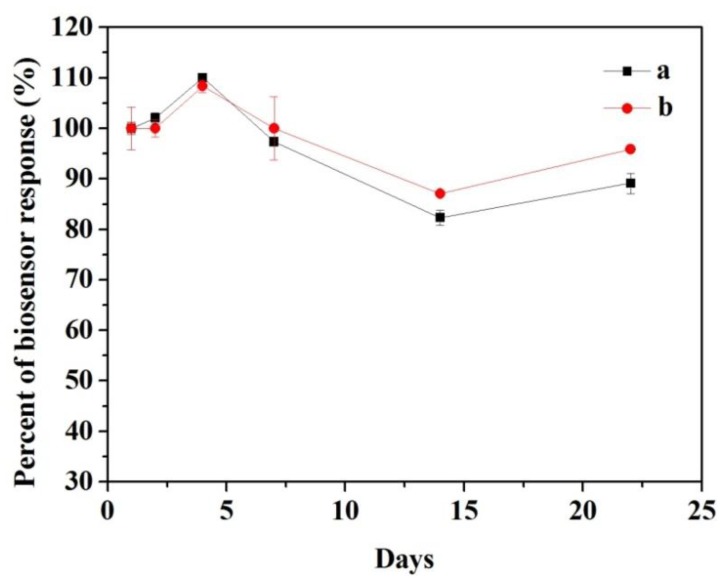
The long-term stability of the response of (**a**) CNFs/Lac/Nafion/GCE and (**b**) Cu/CNFs/Lac/Nafion/GCE. Conditions: scan rate 100 mV s^−1^, pH 5.5(a), pH 6.0 (b). The error bars indicate the standard error for three measurements with the same electrode.

The long-term stability of the biosensors response is presented in Figure 10. The modified electrode was tested three times in 3.92 × 10^−4^ M catechol by cyclic voltammetry to determine how well it performed after extended periods of time. As can be seen in Figure 10a, the response of CNFs/Lac/Nafion/GCE increased to about 110% on the fourth day. The initial increase of the response current resulted from changes of the enzyme's 3D structure inside the immobilization matrix upon rehydration [40]. Thereafter, a sharp decrease was found to continue for another 10 days. Then the response increased from 82.3% to 89.1%, which could be attributed to recovery of the activity of the enzyme by surface renewing [41]. Finally, the biosensor response still retained 89.1% of the initial response after 22 days. While the response of Cu/CNFs/Lac/Nafion/GCE showed a similar change trend, but ended up with 95.9% of the initial response (Figure 10b). These results demonstrated that Cu/CNFs as the immobilized matrix could provide an excellent micro-environment for the stability of laccase. The high stability can be attributed to the capability of entrapping laccase molecules and the good biocompatibility of the carbon-based nanofibers retaining their biological activities.

Under the optimum conditions established for the detection of cathchol in the above section, Guaiacol, vanillin, phenol and 3,5-dinitrosalicylic acid were used to determinate the selectivity of the biosensors via their amperometric responses (Table 1). The concentration of different substrates was 1.96 × 10^−4^ M. It can be seen that vanillin and 3,5-dinitrosalicylic acid almost had no effect on the current responses of the two modified electrodes. Besides, the current response of Cu/CNFs/Lac/Nafion/GCE was lower than that of CNFs/Lac/Nafion/GCE in the detection of guaiacol. Compared to the response of cathchol, these feeble current responses can be ignored. It indicated Cu/CNFs/Lac/Nafion/GCE possessed good selectivity.

It was noted that Cu/CNFs as the immobilized matrix showed better performance than the pure CNFs in liner range, repeatability, reproducibility and stability, which may result from the synergistic effect of the copper nanoparticles and carbon nanofibers. The electrocatalysis and electrical conducting properties of copper nanoparticles and carbon nanofibers showed mutual promotion effects.

## 4. Conclusions

In summary, the biosensing properties of laccase biosensors based on CNFs and Cu/CNFs were studied. Because of good biocompatibility and electrical conducting properties of CNFs and Cu/CNFs, the obtained biosensors when used in the detection of catechol possessed rapid response, a wide linear range, good repeatability, reproducibility, long-term stability and selectivity. It was also found that Cu/CNFs displayed better performance than CNFs as a laccase biosensor. It is expected that Cu/CNFs will have great potential in biosensor construction for environmental monitoring.

## Figures and Tables

**Table 1.** Biosensor response to various substrates.

**Substrate**	**CNFs/Lac/Nafion/GCE**		**Cu/CNFs/Lac/Nafion/GCE**	

	**Current response (μA)**	**Percentage of Response (%)**	**Current response (μA)**	**Percentage of Response (%)**
Catechol	1.9044	100	3.9520	100
Guaiacol	0.6356	33.37	0.1040	2.63
Vanillin	0.0038	0.20	0.0030	0.08
Phenol	0.0036	0.19	0.0620	1.57
3,5-Dinitrosalicylic acid	0.0035	0.18	0.0070	0.17
